# Sarcopenic obesity and associations with mortality in older women and men – a prospective observational study

**DOI:** 10.1186/s12877-020-01578-9

**Published:** 2020-06-09

**Authors:** Å. von Berens, S. R. Obling, M. Nydahl, A. Koochek, L. Lissner, I. Skoog, K. Frändin, E. Skoglund, E. Rothenberg, T. Cederholm

**Affiliations:** 1 Department of Public Health and Caring Sciences, Clinical Nutrition and Metabolism, Uppsala University, Box 561, 751 22 Uppsala, Sweden; 2 Department of Medical Gastroenterology, Odense University Hospital, Odense, Denmark; 3 Department of Food Studies, Nutrition and Dietetics, Uppsala University, Box 560, 751 22 Uppsala, Sweden; 4 Department of Public Health and Community Medicine, Sahlgrenska Academy, University of Gothenburg, Box 453, SE 405 30 Gothenburg, Sweden; 5 Institute of Neuroscience and Physiology, Neuropsychiatric Epidemiology Unit, University of Gothenburg, SU Sahlgrenska, 41345 Gothenburg, Sweden; 6 Kristianstad University, Food and Meal Science, Högskolan Kristianstad, 291 88 Kristianstad, Sweden; 7 Theme Ageing, Karolinska University Hospital, 14157 Huddinge, Sweden

**Keywords:** Older adults, Sarcopenic obesity, EWGSOP2, Prevalence, Mortality

## Abstract

**Background:**

The combined effect of sarcopenia and obesity, i.e., sarcopenic obesity, has been associated with disability and worse outcomes in older adults, but results are conflicting. The objectives of this study were to describe the prevalence of sarcopenic obesity (SO) in older adults, and to examine how the risk of mortality is associated with SO and its various components.

**Methods:**

Data were obtained from two Swedish population studies, the Gothenburg H70 Birth Cohort Studies of 521 women and men at the age of 75, and the Uppsala Longitudinal Study of Adult Men (ULSAM), which included 288 men aged 87 years. Sarcopenia was defined using the recently updated EWGSOP2 definition. Obesity was defined by any of three established definitions: body mass index ≥30 kg/m^2^, fat mass > 30%/ > 42% or waist circumference ≥ 88 cm/≥102 cm for women and men, respectively. The Kaplan-Meier survival curve and the Cox proportional hazard model were used for 10-year and 4-year survival analyses in the H70 and ULSAM cohorts, respectively.

**Results:**

SO was observed in 4% of the women and 11% of the men in the H70 cohort, and in 10% of the ULSAM male cohort. The 75-year-old women with SO had a higher risk (HR 3.25, 95% confidence interval (1.2–8.9)) of dying within 10 years compared to those with a “normal” phenotype. A potential similar association with mortality among the 75-year-old men was not statistically significant. In the older men aged 87 years, obesity was associated with increased survival.

**Conclusions:**

SO was observed in 4–11% of community-dwelling older adults. In 75-year-old women SO appeared to associate with an increased risk of dying within 10 years. In 87-year-old men, the results indicated that obesity without sarcopenia was related to a survival benefit over a four-year period.

## Background

Aging is per se associated with changes in body composition mainly expressed as increase in fat mass, changes in body fat distribution, and loss of muscle mass [1]. The combination of low muscle mass and poor muscle function, i.e., sarcopenia, is a geriatric condition associated with adverse effects on function, quality of life and survival [1–5]. Various definitions of the condition have subsequently been proposed. A decade ago, the European Working Group on Sarcopenia in Older People (EWGSOP) proposed that the combination of low muscle mass and low strength/physical function should define sarcopenia [2]. Recently, the EWGSOP published an updated consensus definition (EWGSOP2) [5]; Sarcopenia was highlighted as a muscle disease, and the new definition emphasizes poor muscle function as the major determinant for the condition rather than low muscle mass.

For middle-aged individuals, the cardio-metabolic risks of overweight and obesity are well established [6]. However, for older individuals there is an ongoing debate concerning the health consequences of obesity, and whether excessive weight might even be beneficial; what is sometimes called the obesity paradox, but data are contradictory [7–9]. There is also controversy as well as a knowledge-gap concerning the impact of body composition, i.e. the proportions of lean and fat mass, on mortality for older adults [10, 11].

There are indications that obesity-related comorbidities vary with age, e.g., optimal body mass index (BMI) values for older adults might be higher than for younger adults [8, 12, 13]. Still, studies also indicate that the risk of physical disabilities increases with obesity in older individuals [14–16].

Given the potential risks related to the two conditions, the combination of the two; i.e. sarcopenic obesity (SO), might be important in its own right [17]. There is an increasing awareness of the potential negative impact of SO in older adults, but results from studies are conflicting [18, 19]. It is reasonable to believe that effects of SO differs with age and sex.

The objective of this study was to use three Swedish cohorts (differing in sex and age) to describe the prevalence of sarcopenic obesity defined by the recently launched EWGSOP2 definition of sarcopenia in combination with any of three common definitions of obesity. A further objective was to examine how the risk of mortality was associated with various body composition phenotypes with a focus on sarcopenic obesity.

## Methods

### Participants

Prevalence figures were calculated by the EWGSOP2 definition for sarcopenia, combined with any of three common definitions for obesity (see Table 1).

**Table 1:** Methods and cut-offs for defining sarcopenia and obesity

	H70 women & men		ULSAM men	
**Sarcopenia** (1)	**Method**	**Cut-off**	**Method**	**Cut-off**
Chair stand (sec)	Five repeated chair stands	> 15 (women & men)	Five repeated chair stands	> 15
Grip strength (kg)	Jamar dynamometer	< 16 (women)	Baseline hydraulic hand dynamometer	< 27
		< 27 (men)		
Muscle Mass, kg/m^2^	BIS	SMI < 5.75(women)/	DXA	ASMI < 7
		< 8.5 (men) (27)		
**Obesity**				
BMI (kg/m^2^)	Balance scale/ standing height	> 30 (women & men) (10)	Balance scale/standing height	> 30
Fat mass (%)	BIS	> 42 (women)	DXA	> 30
		> 30 (men) (28)		
Waist circumference (cm)	Measuring tape^a^	> 88 (women)/ > 102 (men) (29)	Measuring tape^a^	> 102

*BIS* Bioelectrical impedance spectroscopy, *DXA* Dual-energy X-ray absorptiometry, *SMI* Skeletal Muscle Index, *ASMI* Appendicular Skeletal Muscle Index

^a^ measured midway between the lowest rib bone and the iliac crest

To define sarcopenia the updated EWGSOP2 definition was used; i.e. reduced chair-stand capacity (time to perform five repeated chair stands > 15 s) or reduced grip strength (< 16 kg for women and < 27 kg for men), in combination with low muscle mass. Probable or severe sarcopenia [5] was not considered for the SO definition.

In the H70 cohorts, all participants were born in 1930 and data on sarcopenia, obesity, mortality and related covariates for a total of 521 individuals (*n* = 319 women and *n* = 202 men) were collected from the examinations conducted in 2005 when participants were 75 years old (defined as baseline in this study). In the ULSAM cohort, data from 288 community-dwelling men aged 87 years were collected in 2008–2009 (defined as baseline in this study).

### Definitions and cut-offs for sarcopenia, obesity and sarcopenic obesity

Prevalence figures were calculated by using the EWGSOP2 definition for sarcopenia, combined with three different definitions for obesity (Table 1).

The updated EWGSOP2 definition advocates the use of reduced chair-stand capacity (time to perform five repeated chair stands > 15 s) or reduced grip strength (< 16 kg for women and < 27 kg for men) in combination with low muscle mass.

In the H70 Study, bioelectrical impedance spectroscopy (BIS, see below) was used to calculate skeletal muscle mass index (SMI). No cut-offs for SMI are proposed in the EWGSOP2, which is why we chose to use the cut-offs from Janssen et al. for H70 (as in EWGSOP [2] ≤5.75 kg/m^2^ for women and ≤ 8.5 kg/m^2^ for men [20]). In the ULSAM cohort, muscle mass was measured by Dual-energy X-ray absorptiometry (DXA) and the EWGSOP2 recommended cut-off for appendicular skeletal muscle index (ASMI) of < 7 kg/m^2^ was used.

To define obesity, any of three measures of obesity was used, i.e., BMI ≥30 kg/m^2^, fat mass > 42% (women) and > 30% (men), or waist circumference ≥ 88 cm (women) and ≥ 102 cm (men) [6, 21, 22]. If any of the obesity criteria were fulfilled, the individual was defined as having obesity. Individuals defined as having sarcopenia according to EWGSOP2 and concurrent obesity, by any of the definitions, were considered having sarcopenic obesity. SO defined by sarcopenia and elevated fat mass only was used for sensitivity analyses (see below).

### Measurements

#### Body composition

Body composition was measured by BIS using Xitron Hydra 4200 devices (Xitron technologies, San Diego, USA) in the H70 cohorts. Skeletal muscle mass (SMM) from BIS was estimated using the equation (Total Body Skeletal Muscle Mass, no Body weight (TBSMM_noBW)_ = − 24.05 + (0.365*height) + (− 0.005*Ri) + (− 0.012*Re) + (− 1.337*gender)(R_i_ and R_e_ = Intra- and extracellular resistance)) developed and validated by Tengvall et al. [23]. Skeletal muscle index (SMI) was calculated as the ratio of SMM to height in meters squared.

In the ULSAM cohort, DXA (DPX Prodigy, Lunar corp., Madison, WI, USA) was performed and ASMI was calculated using total muscle mass from arms and legs divided by height in meters squared.

#### Strength and function

Grip strength were measured using a Jamar dynamometer in H70 and the Baseline hydraulic hand dynamometer in the ULSAM cohort. The highest value from the strongest hand was used in the analyses, and the thresholds were 16 kg and 27 kg for women and men, respectively. To measure leg strength, the participants were asked (both in H70 and in ULSAM) to perform five repeated chair stands without using their hands. The threshold value for reduced performance was > 15 s [5]. Gait speed, reflecting function, was measured for 30 m indoors at a spontaneously chosen speed in H70. In ULSAM the course was 10 m and the middle six meters were marked and registered.

#### Co-variates

In the regression analyses of body composition phenotypes as exposure for mortality various sets of co-variates were accessible for the two cohorts. In the analyses of the H70 women and men, adjustment was performed for comorbidities and smoking (number of cigarettes/day). Corresponding mortality analyses in the ULSAM male cohort were adjusted for age, comorbidities, education, exercise, living conditions (living alone: yes/no) and smoking (current smoker or non-smoker). When adjusting for co-morbidities, the un-weighted Charlson Comorbidity Index was used in both cohorts. The index was based on in-patient diagnoses (ICD9 - ICD10) in the patient register before the dates of the examinations [24, 25]. In the ULSAM cohort education was assessed by number of years in school divided into categories (7, 8 or 12 years), college education, or graduate exam. Regular exercise was defined as doing sports/heavy gardening more than 3 h per week.

### Statistical analyses

All values are presented as means± SD, median or percentage, as appropriate. In the survival analyses, the cohorts were divided into four groups based on body phenotype: sarcopenic obesity, sarcopenia (without obesity), obesity (without sarcopenia), and no sarcopenia or obesity (i.e. “normal” phenotype) as indicated above. In the analysis of the potential association between SO and all-cause mortality, we examined the 10-year survival in the 75 year old participants of the H70 cohorts (depending on date of examination, maximum years at risk was 9.7) and 4-year survival in the 87 year old participants of the ULSAM cohort (maximum years at risk 4.0). Ten- and four-year observation periods were chosen due to differences in expected survival time in the two cohorts. Analyses were executed using the log-rank test, the Kaplan-Meier survival curve and the Cox proportional hazard model. The Cox regression analyses were presented as hazard ratios with 95% confidence intervals. A *p*-value of < 0.05 was considered statistically significant. Relevant multivariable co-variates for the associations of interest were included in the models. When finding the best fitting model, a likelihood ratio test was performed and a test for proportional hazard assumption including plots of Schoenfeld residuals. All analyses were conducted using STATA15 [26].

#### Sensitivity analyses

In sensitivity analyses (Cox regression for survival), we investigated if the results from the main analyses would remain, both in H70 and in ULSAM, when using only high body fat mass to define obesity (in combination with EWGSOP2 definition for sarcopenia to define SO). Furthermore, we performed sensitivity analyses where the mortality for the women with obesity (no sarcopenia) defined as BMI ≥30 kg/m^2^ was compared to the mortality for the group with no sarcopenia or obesity, and where women with obesity by any of the definitions (irrespective of sarcopenia) were compared to women without obesity.

Also, analyses were performed were individuals within the H70 cohort who had passed away within a year after the examination (2005–2006) were excluded.

The exercise-related co-variate in H70, “*spare time activity during the last year*”, was missing for almost half of the H70 sample. For this reason, complementary sensitivity analyses were performed by adding this co-variate in models that only included individuals with this data available. In the ULSAM cohort, mortality was also compared between the group with obesity (without sarcopenia) defined as waist circumference ≥ 102 cm and those with no sarcopenia or obesity, and between the group with obesity by any definition (irrespective of sarcopenia) and the group without obesity (irrespective of sarcopenia).

Finally, sensitivity analyses (prevalence and cox regression for survival) were also performed using the “original” EWGSOP definition [2].

## Results

Table 2 presents basic characteristics, i.e. anthropometry, body composition and tests of strength and functional performance. In the H70 cohorts, the mean age was 75.6 years for both women and men, and in the ULSAM cohort mean age was 87 years.

**Table 2:** Basic characteristics

	H70 (women) ***n*** = 319	H70 (men) ***n*** = 202	ULSAM (men) ***n*** = 288
Age (yrs)	75.6 ± 0.3	75.6 ± 0.3	86.6 ± 1
Height (cm)	161 ± 6.1	174.9 ± 6.5	172.4 ± 6
Weight (kg)	66.5 ± 10.7	82.2 ± 12.4	74.3 ± 7.8
Body mass index (BMI)(kg/m^2^)	25.7 ± 4.1	26.8 ± 3.6	25.6 ± 3.5
Proportion with BMI ≥ 30 kg/m^2^	20%	15.4%	7%
Body fat mass (BF)(%)	39.7 ± 7.3	31.5 ± 7.5	28.6 ± 7.0
Proportion with BF > 42%	46.6%	62%	44%
Waist circumference (WC) (cm)	86.9 ± 11.5	98.2 ± 10.5	99.6 ± 9.7
Proportion with WC ≥ 88 cm	50%	35%	37%
Skeletal muscle index (SMI) (kg/m^2^)	6.6 ± 0.9	8.6 ± 0.7	7.45 ± 0.8
Grip strength (kg)	24.2 ± 4.3	38.5 ± 7.1	30.2 ± 6.5
Time for five repeated chair stands (sec)	11.9 ± 3.3	12.5 ± 3.9	18 ± 7
Gait speed (m/sec)	1.2 ± 0.2	1.2 ± 0.2	1.36 ± 0.3

In the two cohorts elevated body fat mass was the measure which defined most of the individuals with obesity. In the H70 cohorts, the mean BMI was 26 kg/m^2^ and 27 kg/m^2^ for women and men, respectively. Obesity prevalence by any of BMI, body fat mass or waist circumference was 60% in women and 68% in men in the H70 cohorts. In the ULSAM cohort, average BMI was 26 kg/m^2^, and corresponding obesity prevalence was 55%.

### Prevalence of sarcopenic obesity

#### The H70 cohorts

SO was observed in 4% (*n* = 13) of women and 11% (*n* = 23) of men (Additional file 1, Table S1). For sarcopenia only, i.e. without obesity, the prevalence was around 1% (*n* = 4) for women and < 1% (*n* = 1) for men. Based on the total sample (SO included), 41 subjects (7.8%) were defined as having sarcopenia.

#### The ULSAM cohort

The prevalence of SO was 10%, and the prevalence of sarcopenia only was also 10% (Additional file 1, Table S1).

### Association with mortality

#### The H70 cohorts

**Fig. 1:**
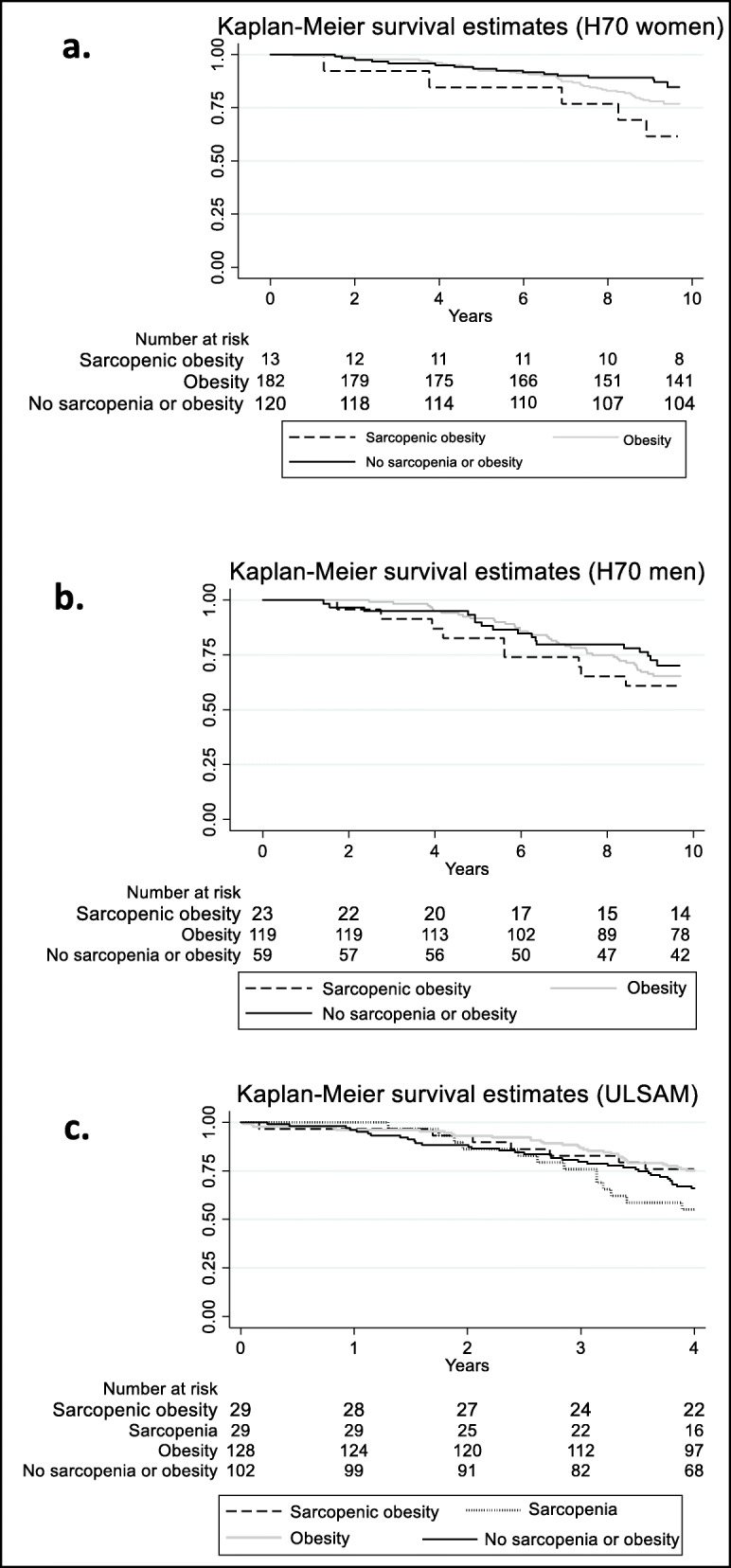
Kaplan- Maier survival estimates according to body composition phenotypes. Panels **a** and **b** show survival rates in women and men from H70 stratified according to “sarcopenic obesity”, “obesity without sarcopenia” and “no sarcopenia or obesity”, adjusted for the covariates comorbidity and smoking. Panel **c** displays corresponding data for ULSAM, adjusted for age, comorbidities, education, exercise, living conditions and smoking. In H70 (women and men), only five were defined as sarcopenic without obesity, and were consequently excluded from the survival analysis

The association with mortality varied with gender, thus the results are presented for women and men separately (Table 3). Since very few were defined as having sarcopenia (*n* = 5) (without obesity), this group was excluded from further analyses. Compared to the group with “normal” body phenotype, i.e., “no sarcopenia or obesity”, the women with SO in the H70 cohort had a three-fold increased risk of dying during the 10 years of follow-up. This result was significant in the crude model, whereas the CIs became wider in the adjusted model (Table 3). The women with obesity only, i.e. without sarcopenia, also had an increased risk (although non-significant) of mortality during the follow-up period compared to the women who had “no sarcopenia or obesity” (Table 3, Fig. 1a). There were only five fatal events among the smaller group of women with SO, whereas among the women with “obesity only” there were 41 fatal events. In the H70 male cohort, no significant association was found between SO and 10-year survival (Table 3), although the pattern of mortality (Fig. 1b) was similar to that of the women. When performing the survival analyses for women and men together (*n* = 521) SO was associated with an increased risk of mortality during the 10 years of follow-up (HR 2.46, 1.3–4.6, crude model). This finding remained significant when adjusted for comorbidities and smoking (HR 2.23, 1.1–4.6).

**Table 3:** Mortality associated with various body composition phenotypes in three cohorts of older women and men

Exposures	Model 1 HR (95% CI)	Model 2 HR (95% CI)	Model 3 HR (95% CI)
**H70 (women)**			
Sarcopenic obesity	3.25 (1.2–8.9)	2.7 (1.0–7.4)	2.6 (0.9–7.2)
Obesity (without sarcopenia)	1.7 (1.0–3.1)	1.6 (0.9–2.9)	1.6 (0.9–2.9)
**H70 (men)**			
Sarcopenic obesity	1.5 (0.7–3.5)	1.5 (0.6–3.5)	1.4 (0.6–3.3)
Obesity (without sarcopenia)	1.2 (0.7–2.1)	1.2 (0.7–2.2)	1.2 (0.7–2.1)
**ULSAM (men)**			
Sarcopenic obesity	0.7 (0.3–1.6)	0.65 (0.3–1.5)	0.8 (0.3–1.9)
Sarcopenia (without obesity)	1.5 (0.8–2.8)	1.35 (0.7–2.6)	1.4 (0.7–2.9)
Obesity (without sarcopenia)	0.7 (0.4–1.2)	0.6 (0.4–1.0)	0.6 (0.3–0.9)

Hazard ratios (HR) and 95% confidence intervals (CI). Ten-year mortality is considered in the H70 studies, while four-year mortality is the outcome in the ULSAM study. Model 1 shows crude analyses in H70 women and men, whereas model 1 adjust for age in ULSAM men. In the two H70 cohorts’ model 2 adjusts for comorbidities, whereas model 2 in the ULSAM cohort includes adjustments for age and comorbidities. Model 3 includes adjustments for comorbidities and smoking in H70, and in ULSAM it adjusts for age, comorbidities, education, regular exercise, living conditions and smoking. The reference group was participants with “no sarcopenia or obesity”

#### The ULSAM cohort

There was no significant difference in survival between participants with SO compared to those with a “normal” body phenotype, i.e. no sarcopenia or obesity (Table 3, Fig. 1c). In the adjusted model, men with obesity only; i.e. without sarcopenia, had a 40% lower mortality risk compared to those with “no sarcopenia or obesity”.

### Sensitivity analyses

#### The H70 cohorts

When obesity was defined exclusively by high amount of body fat (%) the mortality outcome was similar to the previous finding from the main analysis; i.e. women with SO had a three-fold increased risk of dying within the 10 years (HR 3.2 95% CI (1.2–8.8)) compared to those without sarcopenia or obesity. Likewise, this result did not remain significant after adjustment for comorbidity and smoking (Additional file 1, Table S2 and S3).

Moreover, analyses also showed that women with a BMI ≥30 kg/m^2^ (without sarcopenia) had a significantly higher mortality than the women with “no sarcopenia or obesity” although the association did not remain after adjustment for comorbidities. On the other hand, the women with obesity irrespective of sarcopenia displayed increased 10-year mortality even after adjustment for comorbidities and smoking; i.e. HR 1.7 95% CI (1.0–3.0) compared to the women without obesity.

Exclusion of individuals that died within 1 year after baseline or adding the covariate “spare time activities during the last year” to the model did not alter the results.

#### The ULSAM cohort

Defining obesity exclusively by high amount of body fat (%) did not change the result from the main analysis (Additional file 1, Table S2 and S3). When comparing mortality for men with elevated waist circumference ≥ 102 cm to those with “normal” body phenotype (“no sarcopenia or obesity”), somewhat unexpectedly the men with central obesity still had a 40% (HR 0.6, 95% CI (0.4–1.1)) lower risk of dying within the follow-up time (although a wide confidence interval). When adjusted for comorbidities, education, exercise, living conditions, and smoking, this association became even stronger (HR 0.4, 95% CI (0.2–0.8)). When comparing the individuals with obesity by any definition (irrespective of sarcopenia) to those without obesity (irrespective of sarcopenia), results showed a lower risk of mortality in the group with obesity in both the age-adjusted and the fully adjusted model (HR 0.5 95% CI (0.3–0.9)).

#### EWGSOP

When analyses were performed with the use of the original EWGSOP definition [2] for sarcopenia, the results were in accordance with those for EWGSOP2 (Tables S4 and S5).

## Discussion

This study reports on mortality from three cohorts of older adults, different in age and sex, related to sarcopenic obesity, or sarcopenia and obesity separately. One of several interesting results was that 75-year-old women with SO appeared to have at least three times higher risk of dying during the 10 years of follow-up compared to those with “normal” body phenotype (“no sarcopenia or obesity”). No similar association was obvious among the 75-year-old men. In contrast, in the 87-year-old men obesity (irrespective of sarcopenia) appeared to be associated with prolonged survival.

The prevalence of SO for the cohorts in this study is difficult to compare with similar studies, since no studies have so far been published using the EWGSOP2 definition of sarcopenia. In a cross-sectional analysis in an American population using eight different definitions for SO, the prevalence varied up to 26-fold depending on the definition [18]. In a systematic review from 2014, the EWGSOP group reported prevalence’s of sarcopenia of 1–29% using the previous EWGSOP definition from studies of home-dwelling older adults [27]. It is noteworthy that many studies in this field do not distinguish between sarcopenia and sarcopenic obesity. It cannot be ruled out that a proportion of the samples identified as having sarcopenia in previous reports actually displayed sarcopenic obesity. This condition should be considered as a distinct phenotype with specific clinical and metabolic characteristics. This statement is supported by the current finding of increased mortality in the 75-year-old women with SO. However, the present sample sizes were small, and only five fatal events were observed among the women with SO, producing wide confidence intervals. When combining 75-year old women and men the association between mortality and SO became stronger.

Three measures, i.e. BMI, waist circumference and proportion of fat mass, were used to define obesity. Interestingly, the mean BMI in the groups of 75-year-old women with either SO or any type of obesity were below 30 kg/m^2^. In the sensitivity analysis, where mortality was compared between the groups of women with obesity defined as either high BMI only, or high fat mass only, and the women with “no sarcopenia or obesity”, the women with obesity had a higher risk of mortality. However, this association became non-significant after adjustment for co-morbidities. Furthermore, the sensitivity analyses revealed an increased mortality risk in the women with any type of obesity, irrespective of sarcopenia and adjusted for comorbidities and smoking, compared to the women without obesity (irrespective of sarcopenia).

No clear corresponding association between SO or obesity and mortality was found among the 75-year-old men. A possible explanation could be that the health consequences of obesity differ between the genders. The male pattern of obesity is usually more related to increased risks, e.g., the metabolic syndrome, contributing to the fact that men have a shorter life expectancy than women [28]. It is possible, therefore, that some men at increased risk had died before the age of 75. Thus, a selection of men with less metabolically active obesity could have been included in this study. A corresponding selection may not have occurred yet in the 75-year-old women. A study from 2012 examining the relationship between body composition and mortality in Swedish older adults, mean age 72 years, also found a gender difference. However, these data displayed a U-shaped relationship between total fat mass and mortality in men. In women, in contrast to our finding total fat mass was negatively associated with mortality, indicating a protective effect in the women [11].

Although risks associated with obesity are well described in the literature, there is an ongoing debate as to whether this risk weakens with age and whether “the obesity paradox” exists for older adults [16, 37, 38]. In our study, the 87-year-old men with obesity (with or without sarcopenia) appeared to have a lower risk of dying within the 4-year follow-up time, even when obesity was defined as a high waist-circumference only (although not significant). Mechanisms explaining the obesity paradox are not clear, but it is hypothesized that obesity is accompanied by an increase in muscle mass, which could mediate a potential protective effect [9, 29, 30]. Other explanations include that obesity may merely reflect an absence of chronic disease, whereas lower BMI at older age is often associated with chronic catabolic illnesses, triggering unintentional weight loss that contributes to premature death [13, 31].

Reports on risks associated with SO are also conflicting. A recent meta-analysis reported SO to be associated with an increased risk of mortality (24%), especially in men [32]. However, the heterogeneity among the compiled studies was substantial. One of the studies, based on the National Health and Nutrition Examination Survey III (NHANESIII, Batsis et al.), reported results according to gender aspects that were in line with those found in the current study. Thus, the prevalence of SO was higher in men, and SO in women was associated with a higher risk of mortality [33]. In this still young area of research, conflicting results are probably partly due to the heterogeneity of definitions of sarcopenia and SO, as well as the measuring techniques and cut-offs chosen [28, 34].

A general limitation of observational studies, especially when examining older adults, is the risk of selection bias, the “healthy participant effect” [35]. It is reasonable to expect that the older adults that were well enough to participate in the H70 and ULSAM examinations were healthier than the general older population in Sweden. Other limitations of the study include the relatively small sample sizes with few fatal events and a subsequent risk of type 2 errors. The fact that we reported different follow-up times for the two cohorts could be viewed as a short-coming. Still, we found it feasible to have a 10-year follow-up period in the considerably younger participants of the H70 cohorts compared to the 4 years of follow-up in the 87-year old ULSAM cohort. A methodological option could have been to pool the three cohorts and use a meta-analysis approach. Such an approach may have added further perspectives, as well as a mix of cohorts with different ages and gender may also add new uncertainties. Still, such an approach could be an option for future research within this field. Furthermore, most covariates in the cohorts were based on self-reported data (e.g., smoking, education, living alone) and, due to study design, we cannot rule out residual confounding. In the H70 cohorts, due to few events in the group with SO, we limited the inclusion of covariates in the model. The assessment methods, such as that for body composition, were not the same in the various cohorts. Interestingly though, the prediction equation for estimating total body skeletal muscle mass by BIS, used in this study for the two H70 cohorts, has been validated against DXA also using the H70 population with only a small systematic bias being reported [23].

The strengths of this study include the choice of various cohorts, taking gender and different ages (75 and 87 years) into account. As mentioned, pooling of the cohorts had been an alternative design, but the heterogeneity of the populations averted us. Another potential strength is that since BMI has some limitations when used in older populations [29, 30] we chose to include alternative measures of body fat and waist circumference for the assessment of obesity.

## Conclusion

This study illuminates the importance of considering obesity when studying sarcopenia. The results show that SO defined by EWGSOP2 in combination with any of three common measures of obesity, was more prevalent among 75-year-old men than among women of the same age. In contrast to the 75-year-old men, the 75-year-old women with SO seemed to have an increased risk of dying within 10 years compared to women who did not have sarcopenia or obesity. The prevalence of SO in men was higher in the 87-year old’s than in the 75-year old’s, but no association between SO and mortality was found in any of the two groups of men. On the contrary, in the oldest men obesity was associated with improved survival. More studies in this emerging research field, based on larger samples and with special focus on gender and age, are warranted.

## Supplementary information

**Additional file 1 Table S1.** includes basic characteristics for three cohorts of older women and men, groups based on body composition phenotypes. **Table S2** shows the prevalence of sarcopenic obesity, sarcopenia, obesity and no sarcopenia or obesity in three cohorts of older women and men when sarcopenia is defined according to EWGSOP2 and obesity by high body fat. **Table S3** shows mortality associated with various body composition phenotypes in three cohorts of older women and men when sarcopenia is defined according to EWGSOP2 and obesity by high body fat (%), for women > 42% and men > 30%. **Table S4** presents the prevalence of sarcopenic obesity, sarcopenia, obesity and no sarcopenia or obesity in the three cohorts of older adults when sarcopenia is defined according to EWGSOP and obesity as any of the three measures of obesity (BMI, body fat, waist circumference). **Table S5** shows mortality associated with various body composition phenotypes in the three cohorts when sarcopenia is defined according to EWGSOP and obesity as any of the three measures.

## Data Availability

The data used and analysed during the current study are available on reasonable request. The corresponding author could be contacted for further information.

## Abbreviations

ASMI: Appendicular Skeletal Muscle Index
BIS: Bioelectrical Impedance Spectroscopy
DXA: Dual-energy X-ray Absorptiometry
EWGSOP: European Working Group of Sarcopenia in Older People
H70: The Gothenburg H70 Birth Cohort Studies
PPSW: Prospective Population Study of Women in Gothenburg
SMI: Skeletal Muscle Index
SMM: Skeletal Muscle Mass
SO: Sarcopenic Obesity
ULSAM: Uppsala Longitudinal Study of Adult Men
