# Supplementary material for: Sarcopenic obesity and associations with mortality in older women and men – a prospective observational study
Source: BMC Geriatr. 2020 Jun 9;20:199. doi: 10.1186/s12877-020-01578-9 (PMC7285448; doi:10.1186/s12877-020-01578-9)
Supplement: Supplementary file 1 — Additional file 1 Table S1. includes basic characteristics for three cohorts of older women and men, groups based on body composition phenotypes. Table S2 shows the prevalence of sarcopenic obesity, sarcopenia, obesity and no sarcopenia or obesity in three cohorts of older women and men when sarcopenia is defined according to EWGSOP2 and obesity by high body fat. Table S3 shows mortality associated with various body composition phenotypes in three cohorts of older women and men when sarcopenia is defined according to EWGSOP2 and obesity by high body fat (%), for women > 42% and men > 30%. Table S4 presents the prevalence of sarcopenic obesity, sarcopenia, obesity and no sarcopenia or obesity in the three cohorts of older adults when sarcopenia is defined according to EWGSOP and obesity as any of the three measures of obesity (BMI, body fat, waist circumference). Table S5 shows mortality associated with various body composition phenotypes in the three cohorts when sarcopenia is defined according to EWGSOP and obesity as any of the three measures. [file 12877_2020_1578_MOESM1_ESM.docx]

**Supplementary information accompanying this paper.**

Additional files table S1-S5.

**Table S1.** Basic characteristics for three cohorts of older women and men*.

| **H70 (women)** | **Sarcopenic obesity**  **n=13 (4%)** | **Sarcopenia (without obesity)**  **n=4 (1%)** | **Obesity**  **(without sarcopenia)**  **n=182 (57%)** | **No sarcopenia or obesity**  **n=120 (38%)** |
| --- | --- | --- | --- | --- |
| Age (yrs) | 75.7± 0.3 | 75.4± 0.2 | 75.5± 0.4 | 75.6± 0.3 |
| Height (cm) | 162.5± 6.8 | 150.25± 2.8 | 162± 5.9 | 161± 6.1 |
| Weight (kg) | 62.5± 9.9 | 48.8± 1.7 | 74.6± 10.5 | 61.2± 7.4 |
| Body mass index (BMI)(kg/m^2^) | 23.9± 5 | 21.5± 1.0 | 28.5± 3.8 | 23.6± 2.6 |
| Proportion with BMI ≥ 30 kg/m^2^ | 7.7% | 0 | 34.6% | 0 |
| Body fat mass (BF)(%) | 46± 5.9 | 38.1± 1.9 | 43.2± 5 | 35.6± 7.3 |
| Proportion with BF >42% | 84.6% | 0 | 69.8% | 0 |
| Waist circumference (WC) (cm) | 83.8± 12.8 | 70.2± 6.3 | 95.3± 9.5 | 78.5± 6.8 |
| Proportion with WC≥88 cm | 46.1% | N/A | 81.9% | 0 |
| Skeletal muscle index (SMI) (kg/m^2^) | 5.2± 0.8 | N/A | 6.7± 0.8 | 6.6± 0.8 |
| Grip strength (kg) | 19.8± 4.3 | N/A | 25.0± 3.8 | 24.1± 4.2 |
| Time for five repeated chair stands (sec) | 19.7± 9.7 | N/A | 11.8± 1.7 | 11.1± 1.8 |
| Gait speed (m/sec) | 1.01±0.23 | 1.1±0.2 | 1.15±0.2 | 1.2±0.2 |
| **H70 (men)** | **Sarcopenic obesity**  **n=23 (11.4%)** | **Sarcopenia (without obesity)**  **n=1 (0.5%)** | **Obesity (without sarcopenia)**  **n=119 (59%)** | **No sarcopenia or obesity**  **n=59 (29%)** |
| Age (yrs) | 75.4± 0.2 | N/A | 75.6± 0.4 | 75.6± 0.3 |
| Height (cm) | 172.9± 7.7 | N/A | 176± 6.4 | 173.5± 5.5 |
| Weight (kg) | 80.6± 11.1 | N/A | 86.1± 12.7 | 74.9± 8.5 |
| Body mass index (BMI)(kg/m^2^) | 26.9± 3.4 | N/A | 27.4± 3.6 | 24.8± 2.6 |
| Proportion with BMI ≥30 kg/m^2^ | 13% | N/A | 23% | 0 |
| Body fat mass (BF)(%) | 40.3± 4.6 | N/A | 33.6± 5.1 | 23.8± 6 |
| Proportion with BF >30% | 100% | N/A | 83% | 0 |
| Waist circumference (WC)(cm) | 99.8± 9.9 | N/A | 101.3± 9.8 | 91.5± 7.3 |
| Proportion with WC ≥102 cm | 30% | N/A | 52% | 0 |
| Skeletal muscle index (SMI) (kg/m^2^) | 7.8± 0.6 | N/A | 8.6± 6 | 8.9± 0.5 |
| Grip strength (kg) | 32.7± 8.0 | N/A | 39.5± 6.5 | 38.6± 7.5 |
| Time for five repeated chair stands (sec) | 18.4± 5.5 | N/A | 12.1± 3 | 11± 2.7 |
| Gait speed (m/sec) | 1.0± 0.3 | 1.43 | 1.2± 0.2 | 1.3± 0.1 |
| **ULSAM (men)** | **Sarcopenic obesity**  **n=29 (10%)** | **Sarcopenia (without obesity)**  **n=29 (10%)** | **Obesity**  **(without sarcopenia)**  **n=128 (44%)** | **No sarcopenia or obesity**  **n=102 (35%)** |
| Age (yrs) | 86.5± 0.9 | 86.5± 1.0 | 86.6± 1.0 | 86.6± 1.1 |
| Height (cm) | 171.7± 5.4 | 171.1± 7.2 | 172.9± 6 | 172.3 ± 5.8 |
| Weight (kg) | 73.8± 8.1 | 64± 7.9 | 83.7± 9.7 | 70.8± 7.9 |
| Body mass index (BMI)  (kg/m^2^) | 25± 2.4 | 21.8± 2.0 | 28± 2.9 | 23.8± 2.6 |
| Proportion with BMI ≥30kg/m^2^ | 3% | 0 | 14% | 0 |
| Body fat mass (BF) (%) | 33.8± 3.4 | 22.4± 5.3 | 33.3± 4.6 | 23.1± 5.0 |
| Proportion with BF > 30% | 90% | 0 | 78.9% | 0 |
| Waist circumference (WC) (cm) | 100.2± 8.5 | 90.4± 6.9 | 106.5± 7.3 | 93.3± 6.7 |
| Proportion with WC ≥102 cm | 41% | 0 | 75% | 0 |
| Appendicular skeletal muscle index (ASMI) (kg/m^2^) | 6.4± 0.5 | 6.5± 0.4 | 7.8± 0.6 | 7.5± 0.6 |
| Grip strength (kg) | 25.8± 5.3 | 28.1± 5.6 | 30.4± 6.5 | 31.4± 6.5 |
| Time for five repeated chair stands (sec) | 22± 10 | 17± 3 | 18± 7 | 17± 8 |
| Gait speed (m/sec) | 1.2± 0.3 | 1.3± 0.3 | 1.4± 0.3 | 1.4± 0.3 |

*Groups based on body composition phenotypes.

**Table S2.** Prevalence of SO, sarcopenia, obesity and no sarcopenia/obesity in three cohorts of older adults, n (%).

|  | Sarcopenic Obesity | Sarcopenia (without obesity) | Obesity  (without sarcopenia) | No sarcopenia or obesity |
| --- | --- | --- | --- | --- |
| **H70 (women)** | 11(5) | 5(2) | 95(41) | 122(52) |
| **H70 (men)** | 23(13) | 1(1) | 87(51) | 61(35) |
| **ULSAM** | 20(7) | 30(10) | 106(37) | 130(45) |

Sarcopenia is defined according to EWGSOP2 and obesity by **high body fat (%),** for women >42% and men >30%.

**Table S3.** Mortality associated with various body composition phenotypes in three cohorts of older adults.

| Exposures | Model 1  HR (95% CI) | Model 2  HR (95% CI) | Model 3  HR (95% CI) |
| --- | --- | --- | --- |
| **H70 (women)** |  |  |  |
| Sarcopenic obesity | 3.2 (1.2-8.8) | 2.0 (0.7-6.0) | 1.4 (0.6-3.2) |
| Obesity (without sarcopenia) | 1.5 (0.8-2.8) | 1.2 (0.6-2.4) | 1.1 (0.6-1.9) |
| **H70 (men)** |  |  |  |
| Sarcopenic obesity | 1.4 (0.6-3.0) | 1.4 (0.6-3.3) | 1.4 (0.6-3.25) |
| Obesity (without sarcopenia) | 1.1 (0.6-1.9) | 1.1 (0.6-1.9) | 1.1 (0.6-2.0) |
| **ULSAM (men)** |  |  |  |
| Sarcopenic obesity | 0.7 (0.2-1.9) | 0.6 (0.2-1.8) | 0.6 (0.2-2.2) |
| Sarcopenia (without obesity) | 1.6 (0.9-3.0) | 1.5 (0.8-2.8) | 1.7 (0.8-3.7) |
| Obesity (without sarcopenia) | 0.8 (0.5-1.3) | 0.8 (0.5-1.2) | 0.7 (0.4-1.3) |

Hazard ratios (HR) and 95% confidence intervals (CI). Sarcopenia is defined according to EWGSOP2 and obesity **by high body fat (%),** for women >42% and men >30%. Ten-year mortality is considered in the H70 studies, while four-year mortality is the outcome in the ULSAM study. Model 1 shows crude analyses in H70 women and men, whereas model 1 adjust for age in ULSAM men. In the two H70 cohorts’ model 2 adjusts for comorbidities, whereas model 2 in the ULSAM cohort includes adjustments for age and comorbidities. Model 3 includes adjustments for comorbidities and smoking in H70, and in ULSAM it adjusts for age, comorbidities, education, regular exercise, living conditions and smoking. The reference group was participants with “no sarcopenia or obesity”.

**Table S4.** Prevalence of SO, sarcopenia, obesity and no sarcopenia/obesity in three cohorts of older adults, n (%).

|  | Sarcopenic Obesity | Sarcopenia (without obesity) | Obesity  (without sarcopenia) | No sarcopenia or obesity |
| --- | --- | --- | --- | --- |
| **H70 (women)** | 16 (5) | 7(2) | 187(59) | 109(34) |
| **H70 (men)** | 6(3) | 1(0.5) | 139(69) | 56(28) |
| **ULSAM** | 22(8) | 27(17) | 135(48) | 98(35) |

Sarcopenia is defined according to the **EWGSOP** definition of sarcopenia and obesity as any of three measures of obesity, i.e., BMI ≥30 kg/m2, fat mass >42% (women) and >30% (men), or waist circumference ≥88 cm (women) and ≥102 cm (men).

**Table S5.** Mortality associated with various body composition phenotypes in three cohorts of older adults.

| Exposures | Model 1  HR (95% CI) | Model 2  HR (95% CI) | Model 3  HR (95% CI) |
| --- | --- | --- | --- |
| **H70 (women)** |  |  |  |
| Sarcopenic obesity | 1.9(0.7-5.0) | 1.5(0.5-4.5) | 1.5(0.5-4.4) |
| Obesity (without sarcopenia) | 1.5(0.8-2.5) | 1.4(0.8-2.4) | 1.5(0.8-2.6) |
| **H70 (men)** |  |  |  |
| Sarcopenic obesity | 2.3(0.7-7.8) | 1.6(0.4-7.2) | 1.6(0.4-7.3) |
| Obesity (without sarcopenia) | 1.1(0.6-1.9) | 1.1(0.6-1.9) | 1.0(0.6-1.8) |
| **ULSAM (men)** |  |  |  |
| Sarcopenic obesity | 1.1(0.5-2.5) | 1.0(0.4-2.3) | 0.8(0.3-2.4) |
| Sarcopenia (without obesity) | 1.8(0.9-3.3) | 1.5(0.9-2.9) | 1.0(0.4-2.4) |
| Obesity (without sarcopenia) | 0.7(0.4-1.1) | 0.6(0.4-1.0) | 0.4(0.2-0.8) |

Hazard ratios (HR) and 95% confidence intervals (CI). Sarcopenia is defined according to the **EWGSOP** definition of sarcopenia and obesity as any of three measures of obesity, i.e., BMI ≥30 kg/m2, fat mass >42% (women) and >30% (men), or waist circumference ≥88 cm (women) and ≥102 cm (men). Ten-year mortality is considered in the H70 studies, while four-year mortality is the outcome in the ULSAM study. Model 1 shows crude analyses in H70 women and men, whereas model 1 adjust for age in ULSAM men. In the two H70 cohorts’ model 2 adjusts for comorbidities, whereas model 2 in the ULSAM cohort includes adjustments for age and comorbidities. Model 3 includes adjustments for comorbidities and smoking in H70, and in ULSAM it adjusts for age, comorbidities, education, living conditions and smoking. The reference group was participants with “no sarcopenia or obesity”.
